# Wide resection and reconstruction in a low resource area, cemented nail technique knee arthrodesis; a report of case and surgical technique

**DOI:** 10.1016/j.ijscr.2022.107621

**Published:** 2022-09-14

**Authors:** Hassan Mohammed Hassan Elbahri, Hozifa Mohammed Ali Abd-Elmaged, Mohamed Abdulkarim, Mohammed Mubarak Mohammed Ahmed, Mohamed Medani Elhag Medani

**Affiliations:** a International University of Africa, Department of Orthopedics, Sudan; b Alzaiem Alazhari University, Department of Orthopedics, Sudan; c Alzaiem Alazhari University, Department of Surgery, Sudan; d Orthopedics and Trauma, Ibrahim Malik Teaching Hospital, Sudan

**Keywords:** Osteosarcoma, Distal femur, Knee arthrodesis, Cemented nail, Case report

## Abstract

**Introduction and importance:**

Osteosarcomas are primary malignant bone tumors that are driven from bone-forming mesenchymal cells and account for nearly 20 % of primary bone tumors.

**Case presentation:**

A 16-year-old female presented with chief complaint of pain and swelling on her right knee for 6 months with history of trauma. Her knee mobility and ROM was limited due to pain and the mass. Physical examination revealed a 15 × 22 cm mass on distal part of right femur with visible dilated veins. There was normal distal motor, sensory functions. Imaging revealed distal femur mass with mixed lytic and blastic features, wide transitional zone with hair and periosteal reaction; features suggestive of osteosarcoma that was confirmed by histopathological examinations as intramedullary osteosarcoma. She undergone surgical treatment consisting of surgical excision of the mass with safety margins and knee reconstruction by knee arthrodesis using femoral-nail and bone cement technique with excellent outcome.

**Clinical discussion:**

Osteosarcoma is best investigated through plain imaging, MRI and possible CT with histology being confirmatory. It is best approached with meticulous dissection to ensure clear margins or if necessary, amputation. Following resection, reconstruction can be done. In this specific case, the tumor was on the distal femur and the underlying knee was arthrodesed using cemented nail technique in which a cemented intramedullary nail was inserted with excellent clinical outcome.

**Conclusion:**

Surgical approach to osteosarcoma can be performed through limb salvage or amputation. Arthrodesis with cemented nail technique using an intramedullary nail can be performed in some patients with excellent clinical outcome.

## Highlights

• Osteosarcomas are malignant bone tumors driven from bone-forming mesenchymal cells.
• Preoperative imaging usually performed: plain radiographs, MRI and possible CT scans.
• Best approached through meticulous dissection or if necessary, through amputation.
• Arthrodesis can be used for limb salvage which is well-known for centuries.

## 1 Introduction

Osteosarcomas is a primary malignant bone tumor that is driven from bone-forming mesenchymal cells and accounts for nearly 20 % of all primary bone tumors [1]. Although, it is a disease that has been present for so long and there are many advances in the treatment, the etiology remains unclear with many factors are suspected to be involved including genetic and environmental factors [2].

Based on the location of the lesion, osteosarcomas can be divided into central (intramedullary), surface (periosteal/cortical) and extra skeletal [3].

Osteosarcomas usually present insidiously resulting in patients presenting weeks to months from when the tumor has actually started forming. Patients usually present with symptoms of bone pain especially with activity. Given the specific age group it is more common in (i.e., second decade of life), parents usually attribute that pain to growing pains, arthritis or even simple sprains in case of associated traumatic events. Pathological fractures are not common with osteosarcomas [4].

## 2 Patient information

A 16 years old female who is a high school student presented with a chief complaint of pain and swelling on her right knee for 6 months. She has history of a fall while playing. She had difficulty with mobilization due to large mass and pain. She performed daily activities and ambulation with the assistance of her family. She has been taken to a traditional bone setter several times. There was no history of allergies or history of the malignancy in her family.

## 3 Clinical findings

The physical examination revealed a mass on the distal part of the right femur, visible dilated veins over swelling, no distal oedema with the knee in a flexed position. The size of the mass was 15 × 22 cm. The mass was immobile with an ill-defined border. It was hot compared to the surrounding and contralateral parts with normal distal motor, sensory and distal capillary refill time. There were intact popliteal, dorsalis pedis and posterior tibial arteries pulsations. The knee range of motion was limited to 100°. There was no inguinal lymphadenopathy.

## 4 Timeline

The patient started complaining of the above-mentioned symptoms 6 months prior to presentation after her trauma, a period during which she visited a traditional bone setter with no relief. Instead, the tumor has even increased in size within the last 3 months. Once admitted, immediate investigations followed by treatment were commenced.

## 5 Diagnostic assessment and interpretation

Laboratory examination revealed normal inflammatory markers. The hemoglobin level was 9.1 g/dL, leucocytes count was 8.7/μL, alkaline phosphatase was 500 U/L; lactate dehydrogenase was 157 U/L. Radiographs showed a distal femur with mixed lytic and blastic lesion, wide transitional zone with hair and end periosteal reaction. The MRI revealed a heterogenous mass at the distal femur (Low T1 and high T2) with no skip lesion and minimal soft tissues extension. The lesion did not affect the proximal part of the tibia. There was no neurovascular bundle involvement based on the MRI. A bone scan and CT chest revealed no distal metastasis. The lesion was staged as grade IIB according to Enniking classification [5].

She was then planned for core needle biopsy and the histopathology revealed malignant cells producing osteoid and stromal cells that are highly malignant with increased nuclear cytoplasmic ratio, hyperchromasia and cellular pleomorphism features pointing towards the diagnosis of conventional intramedullary osteosarcoma.

## 6 Intervention

**Fig. 1:**
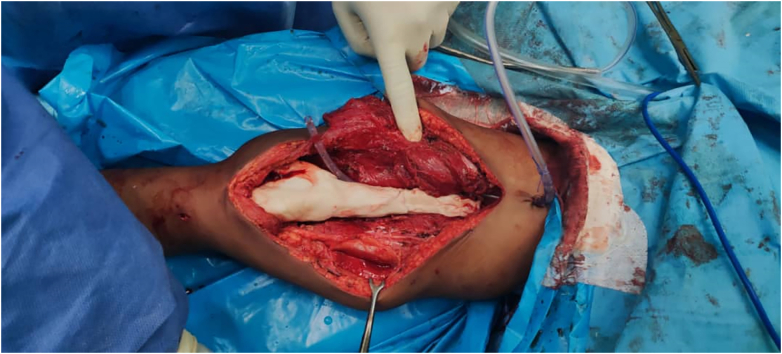
Gross image for the cemented bone.

**Fig. 2:**
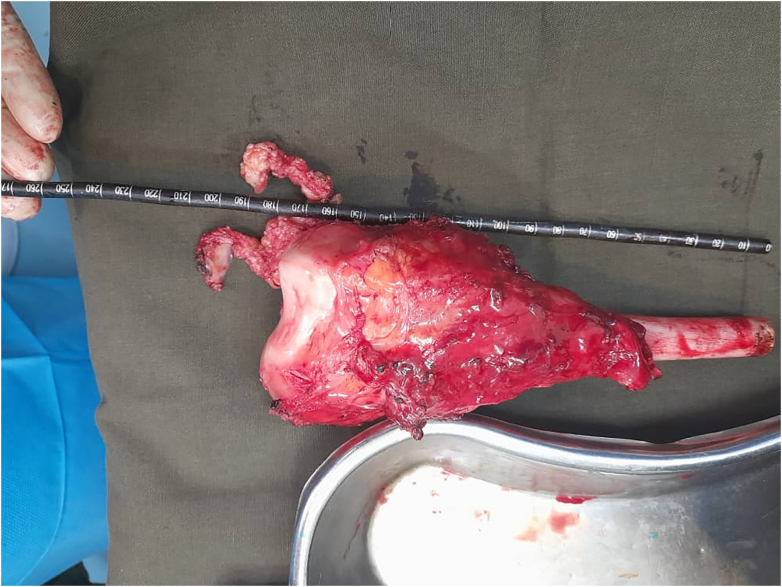
Gross image for the resected mass from the distal part of the fibula.

The surgery performed consisted of wide excision of the mass with reconstruction by knee arthrodesis using femoral-nail and bone cement technique (Fig. 1, Fig. 2). Pre-operatively, the patient was set in a supine position with sterile preparation and draping done.

The skin was incised and the mass was dissected until the tumor was exposed. Fortunately, the neurovascular bundle in the popliteal area has not been not affected by the tumor. Nonetheless, the tumor was already affecting the joint; therefore, the distal 24 cm of the femur was cut to ensure safety margins, and the tumor was removed by intraarticular resection.

After the tumor was excised, the defect was 32 cm from the distal resected femur to the proximal tibia. The cement arthrodesis was then performed. First, the femoral-nail was inserted from the distal femur into the proximal tibia and the length of the nail that is not inserted into the long bone is 32 cm to ensure the same length as the contralateral leg.

**Fig. 3:**
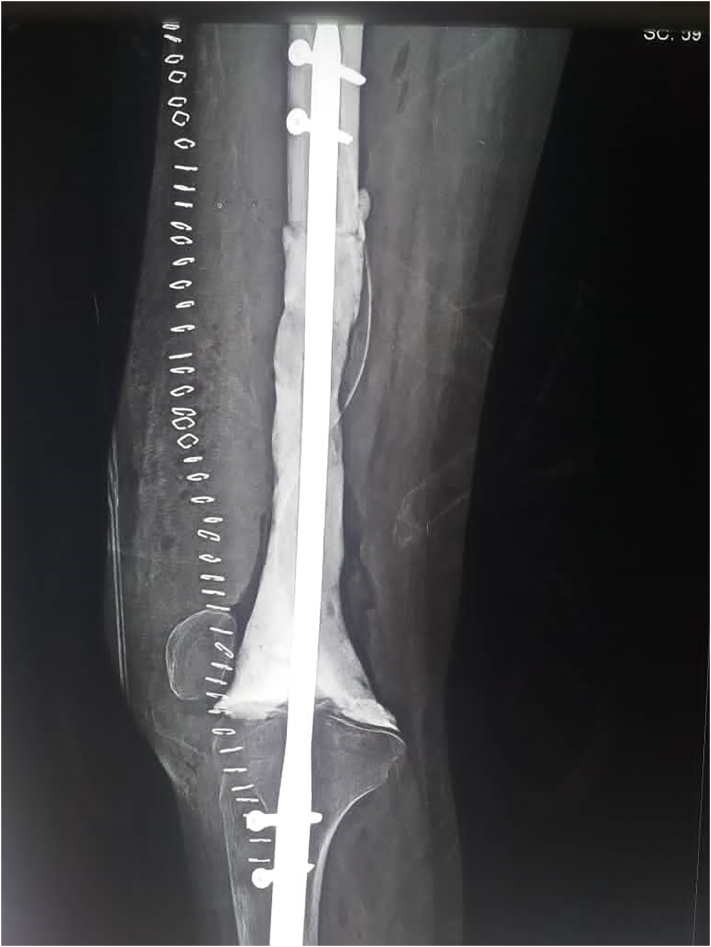
Showing the nail, 4 screws and bone cement used to fill the defect between the bone ends.

We modified the nail direction to ensure a 3° degrees valgus in arthrodesed knee and to allow the anterior to posterior direction of screws to facilitate proximal interlocking screws insertion without repositioning the patient. The nail was then fixed with 2 proximal and 2 distal interlocking screws. The bone cement was augmented to fill the defect between the bone ends (Fig. 3). The length was also evaluated and equalized with the contralateral limb. When we faced a small bone with a very small and narrow canal, we used interlocking nail humerus as a tool for arthrodesis. The soft tissue reconstruction was performed to cover the prosthesis.

This surgery was done by a consultant orthopedic oncologist assisted by an orthopedic oncology trainee.

## 7 Follow-up and outcome

The resected part of tumor was sent to histopathology which revealed clear surgical margins. The patient underwent an uneventful postoperative recovery period with starting of walking using cane from day one. There were no complications related to the wound.

## 8 Discussion

Osteosarcoma is a malignant primary bone tumor of mesenchymal origin. It commonly affects and has a peak incidence in young adult patients and adolescents [6]. Most cases are sporadic in nature and about 70 % of cases have some genetic mutations, most commonly affecting tumor suppressor genes or DNA helicases [7].

To diagnose any suspicious bone mass, a pre-operative imaging is usually performed consisting of plain radiographs, MRI and possible CT scan. A two views X-ray will usually show a mass with non-specific findings of an ill-defined lesion with osteoblastic or mixed osteoblastic/osteoclastic features and a periosteal reaction [8].

Osteosarcoma is generally treated with combining surgery and chemotherapy whether it is neoadjuvant or adjuvant or both [9], [10]. When performing the surgery, the aim usually is resecting the tumor with wide and safety margins. Surgical options for treating osteosarcomas affecting the limbs are either limb salvage which is the method used for up to 85–90 % of patients with 2 steps of resection and reconstruction or amputating the affecting limb [11].

One and not commonly practiced procedure for limb salvage is knee arthrodesis which is a procedure that is well-known and practiced for centuries and has some traces of radiological evidence been found in an Egyptian mummy. It can now be used among other modalities in some conditions including failed total knee arthroplasty as a modality for limb salvage, reconstruction of the knee after removal of a tumor as well as any condition in which there is an inability to reconstruct the knee as in severe trauma or in traumatic osteoarthritis in young patients [12].

Many approaches have been described with the shared goal of maintaining the knee stability including external fixation with Ilizarov apparatus, internal fixation using a plate as well as intramedullary nailing [13], [14], [15] with a widely variable complication rate (20 % to 84 %) [16]. While each method has its own pros and cons such as the prolonged course of treatments as well as cosmetic and functional outcomes for external fixation and increasing risk of wound problems as a result of putting much stress on the already damaged structures in internal fixation, intramedullary nailing permits full weight bearing with higher rates of fusion when compared to external fixation [17].

When comparing this specific case to previous cases, there are some similarities to a previous most recent case of Achmad Fauzi Kamal et al. [18] in which they have managed an 18-year-old male patient with a huge chondromyxoid fibroma of proximal tibia with a cement arthrodesis of his knee using an intramedullary nail. Based on their outcome for this specific case and according to the literature they have described, they suggested that cement arthrodesis provides less time for surgery, lesser infection rate as well as good clinical outcomes. In a wider scale, R Capanna et al. [19] have been able to do resection of the tumor followed by cement arthrodesis in 76 patients with distal femur or proximal tibia tumors with patients being able to bear weight within only days after their surgeries with a 14 % infection rate and 16 % failure of implants rate through fracture or intramedullary Küntscher rod position changes. They have described that cement arthrodesis can help avoid the unpreferable side effects of chemotherapy on graft incorporation.

## 9 Conclusion

The learning objective is that osteosarcoma is malignant bone tumor of mesenchymal origin that usually affects patients in their second decade of life and is best approached through meticulous dissection to ensure clear and safe margins or if necessary, amputation can be performed. Following the resection, reconstruction can be done. In this specific case, the tumor was on the distal femur and the underlying knee was arthrodesed using cemented nail technique in which a cemented intramedullary nail was inserted with excellent clinical outcome for a 16-year-old female patient. This technique, although it has been known before, is not commonly used due to advances in care provided to patients. Here, however, we have done such procedure due to limited facilities and the economic status of the patient and we think this could be a bridge till eventual and final treatment can be provided.

This case has been reported in line with the SCARE criteria [20].

## Consent

Written informed consent was obtained from the patient for publication of this case report and accompanying images. A copy of the written consent is available for review by the Editor-in-Chief of this journal on request.

## Ethical approval

No ethical approval was required for this case report and only informed consent was taken from the patient.

## Funding

Authors received no funding from any source and this work is completely a voluntary work.

## Provenance and peer review

Not commissioned, externally peer-reviewed.

## Author contribution

Hassan Mohammed Hassan Elbahri: Involved in study design, data acquisition, drafting the article and revising it critically and finally approved the manuscript.

Hozifa Mohammed Ali Abd-Elmaged: Involved in study design, data acquisition, drafting the article and revising it critically and finally approved the manuscript.

Mohamed Abdulkarim^3:^ Involved in study design, data acquisition, drafting the article and revising it critically and finally approved the manuscript.

Mohammed Mubarak Mohammed Ahmed: Involved in study design, data acquisition, drafting the article and revising it critically and finally approved the manuscript.

Mohamed Medani Elhag Medani^:^ Involved in study design, data acquisition, drafting the article and revising it critically and finally approved the manuscript.

## Research registration number

Not applicable.

## Guarantor

Hozifa Mohammed Ali Abd-Elmaged.

## Declaration of competing interest

Authors report no conflict of interest of any sort.
